# Exploratory Evaluation of Combined RENAL Nephrometry and Mayo Adhesive Probability Scores for Perioperative Outcomes in Radical Nephrectomy

**DOI:** 10.3390/diagnostics16111647

**Published:** 2026-05-27

**Authors:** Mahdi Alayyafi, Omar Safar, Adel Elatreisy, Saad Thamer, Nazal A. Almasoud, Mohammed Alsharif, Hussain Munyif, Mohammed A. Alshehri, Mohammad Abdelmoaty, Ibrahim Abdel-Al, Sultan Albarman, Mahmoud Z. El Madawie, Mohammed F. Bazeed, Abdulhadi AlQahtani, Abdulrahman Al-Aown

**Affiliations:** 1 Urology Department, Armed Forces Hospital, Khamis Mushayt 62431, Saudi Arabia; mahdiala20@gmail.com (M.A.); saadkkumed@gmail.com (S.T.); s.borman93@gmail.com (S.A.); mahmoudmadawy@gmail.com (M.Z.E.M.); ahadikahtani@yahoo.com (A.A.); aown22@hotmail.com (A.A.-A.); 2 Urology Department, Faculty of Medicine, Al-Azhar University, Cairo 11754, Egypt; dr_adelelatreisy@yahoo.com; 3 Urology Department, King Fahd Armed Forces Hospital, Jeddah 23311, Saudi Arabia; 4 Urology Department, Prince Mutaib bin Abdulaziz Hospital, Sakaka 72341, Saudi Arabia; nazalahmad88@gmail.com; 5 Urology Department, Aseer Central Hospital, Abha 62523, Saudi Arabia; alsharif517@gmail.com; 6 Urology Department, King Khalid Hospital, Najran City 66262, Saudi Arabia; hussainmunief@gmail.com; 7 Radiology Department, Armed Forces Hospital, Khamis Mushayt 62431, Saudi Arabia; mas3399@hotmail.com (M.A.A.); m_bazeed@yahoo.com (M.F.B.); 8 General Surgery Department, Armed Forces Hospital, Khamis Mushayt 62431, Saudi Arabia; dr.moha.abdelmoaty@gmail.com; 9 Urology Department, Faculty of Medicine, Al-Azhar University, Assiut Branch 71524, Egypt; dribrahemuro2011@yahoo.com

**Keywords:** RENAL, Mayo adhesive probability, radical nephrectomy

## Abstract

**Background**: The RENAL Nephrometry Score evaluates renal tumor complexity, while the Mayo Adhesive Probability (MAP) score predicts Adherent Perinephric Fat during nephrectomy. Although both are useful in partial nephrectomy planning, their role in radical nephrectomy (RN) remains unclear. This study assessed whether combining the RENAL and MAP scores improves the prediction of perioperative outcome after RN. **Methods**: In this retrospective study, 52 patients who underwent radical nephrectomy for presumed renal malignancy at a tertiary care hospital in Saudi Arabia between January 2024 and June 2025 were analyzed. RENAL and MAP scores were calculated from preoperative CT scans. Primary outcomes included intraoperative complications, operative time, and length of hospital stay. Associations were evaluated using Spearman correlation, ROC curve analysis, and logistic regression. Given the limited sample size and low event rate, all analyses were considered exploratory. **Results**: Only clinically significant complications, classified as Clavien–Dindo Grade IIIb, were observed, representing 11.5%. Neither RENAL nor MAP scores, alone or combined, showed significant associations with intraoperative complications, blood loss, or operative duration. The RENAL score demonstrated a modest correlation with length of hospital stay (r = 0.294, *p* = 0.035), whereas the MAP score showed a weaker, non-significant correlation (r = 0.191, *p* = 0.175). ROC analysis indicated limited predictive performance for complications (RENAL AUC = 0.377, *p* = 0.330; MAP AUC = 0.732, *p* = 0.067; combined AUC = 0.638, *p* = 0.276). Logistic regression revealed that neither score nor key clinical variables were independent predictors. **Conclusions**: RENAL and MAP scores were not statistically significantly associated with perioperative outcomes in this exploratory cohort.

## 1. Introduction

Renal cell carcinoma (RCC) is the sixth most common cancer in males and the tenth most common cancer in females worldwide, accounting for 5% of all cancers in men and 3% in women [1]. The increase in incidentally detected cancers on radiological imaging, such as ultrasonography and computerized tomography (CT), has contributed to this phenomenon [2]. Although radical nephrectomy (RN) was previously considered the established therapy for renal cell cancer [3], there has been a significant increase in the use of partial nephrectomy (PN) over the past few years. This trend can be attributed to its benefits, including greater preservation of renal function, shorter hospital stays, and reduced intraoperative hemorrhage.

Moreover, multiple studies have demonstrated that PN is superior to RN in overall survival, cancer-specific survival, and recurrence-free survival [4,5]. Additionally, the procedure is both labor-intensive and technically demanding when addressing complex kidney malignancies [6]. The principal concern during surgery is the occurrence of intraoperative complications, which can happen in up to 13.5% of cases [7].

Various grading methods for characterizing the architecture of renal masses have been developed to assist in selecting appropriate surgical procedures and enhancing the prediction of complications associated with renal malignancy. The RENAL (radius, exo-/endophytic characteristics, nearness, anterior/posterior, relative to polar lines) score has gained widespread acceptance due to its straightforward calculation and high level of consistency [8]. Complexity is categorized as low (4–6 points), moderate (7–9 points), and high (10–12 points). Nevertheless, Adherent Perinephric Fat (APF) constitutes another significant feature that has historically been overlooked. APF is closely associated with surgical duration, estimated blood loss volume, and the need to convert to radical surgery [9,10,11,12].

APF presents a challenge for urologists undertaking partial nephrectomies (PNs) or radical nephrectomies (RNs). Commonly referred to as “sticky fat” or “toxic fat,” APF denotes visceral fat that adheres to Gerota’s fascia and contacts the renal parenchyma. The presence of APF complicates kidney mobilization and tumor exposure during partial nephrectomies, thereby increasing procedural complexity [11,12,13]. The Mayo Adhesive Probability (MAP) score, originally introduced by Davidiuk et al. [14], is a radiologic classification scale used to predict the likelihood of Adherent Perinephric Fat formation during radical nephrectomy. The MAP score is calculated based on two parameters: the thickness of posterior perinephric fat and the presence of stranding. While these scoring systems have been extensively examined in partial nephrectomy, their applicability to radical nephrectomy remains less clearly defined because of differences in surgical complexity and complication profiles.

Few studies have examined these two scores in partial nephrectomy, and no studies have evaluated their combination in radical nephrectomy. The objective of this investigation was to assess the predictive utility of the Mayo Adhesive Probability (MAP) score, in conjunction with the RENAL score, concerning perioperative outcomes in cases of radical nephrectomy (RN).

## 2. Methods

This retrospective study included all consecutive patients who underwent radical nephrectomy for presumed renal malignancy at our institution between January 2024 and June 2025, regardless of final histopathological diagnosis, provided that preoperative contrast-enhanced CT imaging was available for scoring during this period. The study was approved by the Institutional Review Board (835).

Given the single-center design and the objective of providing a preliminary assessment of RENAL and MAP scores (individually and in combination) in the understudied context of radical nephrectomy, the available cohort maximized statistical power by leveraging existing institutional data without requiring prospective recruitment. However, with only 6 Grade IIIb complications observed (an approximately 11.5% event rate), logistic regression and ROC analyses were conducted in an exploratory manner; thus, the results should be interpreted with caution.

### 2.1. Data Collection

We collected the primary baseline characteristics of the patients, including age, tumor laterality, associated comorbidities (e.g., diabetes and hypertension), computed tomography (CT) findings, tumor histopathology, and procedures performed. Additionally, we collected outcomes of interest, including complications, mortality, hospital stay duration, and blood loss. To calculate the RENAL score, data were collected on the following variables: renal mass size, renal mass radius, exophytic/endophytic nature, proximity to the collecting system or sinus measured in millimeters, anterior/posterior positioning, placement relative to the polar lines, and the presence of a hilar tumor. All scores were retrospectively determined from preoperative CT images by two investigators. Furthermore, data on lateral peripheral fat thickness, posterior distance, posterior fat score, and stranding score were collected to compute the MAP score. The RENAL and MAP scores were independently calculated by two urologists based on preoperative contrast-enhanced CT images.

### 2.2. Statistical Analysis

All analyses and calculations were conducted utilizing the Statistical Package for the Social Sciences (SPSS, version 26, IBM Corp., Chicago, IL, USA). The normality of continuous variables was assessed using the Kolmogorov–Smirnov test. Data are expressed as means ± standard deviations for continuous variables. Fisher’s exact test was employed to evaluate the association between complications and RENAL and MAP scores. The Spearman correlation coefficient was applied to examine the relationships between RENAL and MAP scores and variables such as blood loss, operative time, and length of hospital stay. The Mann–Whitney U test was used to assess associations between complications and variables such as blood loss, operative time, and length of hospital stay. The area under the receiver operating characteristic curves (AUC) was calculated to evaluate predictive performance. For the combined model, binary logistic regression was used with both RENAL and MAP scores entered as continuous independent predictors; the predicted probability from this model was used to generate the combined ROC curve. A *p*-value of less than 0.05 was considered statistically significant. Given the low number of outcome events (n = 6), multivariable modeling is highly susceptible to overfitting, and the resulting estimates should not be interpreted as reliable inferential findings; all regression and ROC analyses were considered exploratory and hypothesis-generating. Confidence intervals were estimated using the Hanley and McNeil method due to the limited sample size and the absence of bootstrapping. No internal or external validation of predictive models was performed due to sample size constraints. This study is reported in accordance with the STROBE guidelines for observational studies.

### 2.3. Bias Considerations

To minimize potential sources of bias in this retrospective study, several measures were implemented. Selection bias was addressed by including all consecutive patients who met imaging and procedural criteria, regardless of final histopathological diagnosis, and underwent radical nephrectomy during the specified period (January 2024 to June 2025) at our single institution. There were no additional exclusion criteria beyond a confirmed RCC diagnosis and availability of preoperative contrast-enhanced CT imaging suitable for scoring. Information bias (misclassification) in the RENAL and MAP score calculations was reduced by having two independent urologists (O.S. and N.A.A.) perform blinded, retrospective scoring of standardized preoperative CT images; discrepancies were resolved by consensus review. Although the scores were independently evaluated by two reviewers and resolved by consensus, interobserver agreement statistics (e.g., kappa or intraclass correlation) were not calculated, representing a limitation. Outcome ascertainment (intraoperative complications, blood loss, operative time, and length of hospital stay) relied on prospectively documented electronic medical records and operative reports, with Clavien–Dindo grading applied uniformly by the reviewing team. No formal adjustment for unmeasured confounding was performed due to the study’s exploratory nature and the small event rate, but potential confounders (age, diabetes, and hypertension) are reported descriptively and were included in logistic regression models where feasible.

### 2.4. Outcome Definition

The primary endpoint was clinically significant perioperative complications, defined as Clavien–Dindo Grade IIIb events occurring during the index hospitalization or within 30 days postoperatively. The Clavien–Dindo classification was employed to standardize complication severity. Mortality was characterized as perioperative death and is reported separately.

## 3. Results

The study cohort included 52 individuals, with an average age of 51.64 ± 16.12 years. The prevalence of diabetes and hypertension among the participants was 38.5% and 50.0%, respectively. All observed complications were classified as Clavien–Dindo Grade IIIb; no minor (Grade I–II) or other major (Grade IIIa–V) complications were recorded. Six patients (11.50%) reported Grade IIIb complications; no additional complications were documented. The six Grade IIIb complications included [e.g., postoperative hemorrhage requiring surgical re-exploration (n = 2), urinary leak requiring intervention under general anesthesia (n = 1), wound complications requiring operative management (n = 1), and other procedure-related reinterventions (n = 2)]. Given the limited number of events, subgroup comparisons and regression analyses were performed with caution. Masses were classified as moderate complexity in 28 cases (53.8%) and high complexity in 24 cases (46.2%). Additionally, based on the MAP score, 22 cases (42.3%) were classified as moderate probability and 18 cases (34.6%) as high probability (Table 1).

This study compares RENAL and MAP scores by complication status. A total of 66.7% of patients had a moderate RENAL score, whereas 33.3% had a high score; there was no statistically significant difference (*p* = 0.674). Conversely, among patients without complications, 52.2% had moderate scores, and 47.8% had high scores. Comparatively, 33.3% of patients with complications demonstrated moderate MAP scores, whereas 66.7% exhibited high scores; again, no significant difference was observed (*p* = 0.172). Among patients without Grade IIIb complications, 26.1% had low scores, 43.5% had moderate scores, and 30.4% had high scores (Table 2).

The RENAL score demonstrated a negative correlation with blood loss (r = −0.114) and positive correlations with operative time (r = 0.026) and length of hospital stay (r = 0.294). The MAP score was positively correlated with blood loss (r = 0.207), surgical duration (r = 0.211), and length of hospital stay (r = 0.191). When stratified by surgical approach, operative time and blood loss appeared numerically higher in open procedures compared to laparoscopic cases; however, formal statistical comparison was limited by sample size. Nonetheless, no statistically significant correlations were identified, except for the association between length of hospital stay and the RENAL score (Table 3).

No statistically significant differences were observed between patients with and without Grade IIIb complications regarding operative time, blood loss, length of hospital stay, RENAL score, or MAP score (Mann–Whitney U test; Table 4).

This analysis presents a binary logistic regression analysis of the relationship between Grade IIIb complications and variables, including age, diabetes mellitus, hypertension, operative duration, blood loss, length of hospital stay, RENAL score, and MAP score. None of these factors emerged as significant predictors of complications, with *p*-values exceeding 0.05. Notably, age (OR = 0.852, *p* = 0.075) approached statistical significance, indicating a potential, albeit unconfirmed, association. Diabetes mellitus, hypertension, operative time, length of hospital stay, RENAL score, and MAP score did not demonstrate significant predictive value. There is no significant impact on the risk of complications (Table 5). The broad confidence intervals observed in the regression analyses indicate model instability attributable to a low events-per-variable ratio, thereby constraining the reliability of multivariable estimates.

Receiver operating characteristic (ROC) analysis demonstrated limited discriminatory performance. The AUC for the RENAL score was 0.377 (95% CI: 0.16–0.59), the MAP score was 0.732 (95% CI: 0.52–0.94), and the combined model yielded an AUC of 0.638 (95% CI: 0.41–0.86). None of the models reached statistical significance. Given the small number of outcome events (n = 6), the confidence intervals were wide and should be interpreted with caution. Nonetheless, no patient demonstrated a statistically significant capacity to predict complications (Table 6).

## 4. Discussion

The present study aimed to investigate the effectiveness of combining RENAL and MAP scores to predict complications and to examine their correlations with operative time, blood loss, and length of hospital stay following radical nephrectomy. Nevertheless, our findings differ from those of prior investigations regarding the relationship between these scores and the incidence of complications; specifically, this study showed no significant difference in complication incidence across the RENAL and MAP score categories (low, intermediate, and high). The inclusion of both open and laparoscopic radical nephrectomy introduces procedural heterogeneity, which may influence perioperative outcomes and obscure potential associations between nephrometry scores and complications. The absence of statistically significant associations in this cohort should not be interpreted as evidence of no effect, but rather as a reflection of limited statistical power and low event rates, which may have increased the risk of Type II error.

In contrast, earlier studies have documented a statistically significant association between higher RENAL and MAP scores and an increased incidence of complications compared to lower scores [15,16]. The discrepancy between our findings and those of prior studies may reflect key differences in the surgical context, as most prior studies evaluated partial nephrectomy, in which anatomical complexity and perinephric fat adherence may play more prominent roles in determining outcomes. Findings from partial nephrectomy studies may not be directly applicable to radical nephrectomy due to differences in surgical complexity and complication profiles. This discrepancy may be attributed to variations in the reported complications and their definitions across studies, as well as the limited sample size in our study, which may have hindered our ability to achieve statistical significance. Additionally, the use of RN in the present study, unlike nephron-sparing surgery, is associated with a distinct complication rate. Furthermore, Jin et al. [15] reported that these scores correlated with estimated blood loss and operative duration in a study of 293 patients; however, our findings did not reach statistical significance. Nonetheless, the RENAL score was positively associated with hospital stay duration in the present study.

While traditional nephrometry tools such as the RENAL and MAP scores were originally developed and validated primarily in the context of partial nephrectomy, recent work has emphasized the potential of more refined CT-derived morphometric signatures to guide intraoperative decision-making. Preoperative CT-based parameters, including tumor–parenchyma contact surface area (CSA > 15 cm^2^), tumor radius, and Gerota’s fascia thickness, independently predict the necessity for renorrhaphy versus a sutureless reconstructive strategy during off-clamp minimally invasive partial nephrectomy [17]. These radiologic markers improved the RENAL score’s performance (ΔAUC +0.06; NRI 0.14; IDI 0.07), achieving strong model discrimination (AUC 0.81) [17]. Although developed for partial nephrectomy, these CT signatures might enhance preoperative risk assessment in radical nephrectomy and warrant validation in this group.

The surgical management of renal cell carcinoma (RCC) is a complex and challenging endeavor influenced by various factors, including the surgeon’s expertise, tumor characteristics, and the presence of Adherent Perinephric Fat (APF) [7,17]. The RENAL Nephrometry Score is widely acknowledged and frequently employed to evaluate anatomical features [8]. Nevertheless, these assessment tools predominantly emphasize tumor anatomic traits, often neglecting the tumor environment. Historically, anatomical measures such as the RENAL score have been the primary means for evaluating surgical complexity. However, in recent years, Adherent Perinephric Fat (APF)—which can be identified using the MAP score—has been recognized as a significant, yet previously overlooked, contributor to adverse outcomes [11,12]. These two scoring systems are distinct and specifically designed to target different surgical procedures.

The present study demonstrated a low incidence of complications, affecting only six patients (11.5%), consistent with earlier research [7,14,15,18,19]. This low rate may be attributed to the efficacy of radical nephrectomy (RN). Conversion from nephron-sparing surgery to radical nephrectomy is occasionally necessitated in challenging cases, such as when positive surgical margins are present, bleeding control is inadequate, infiltration into the collecting system occurs, or technical limitations exist; however, it is generally considered undesirable. The reported conversion rate ranges from 5.0% to 13.5% [7,18,20,21]. Jin et al. [15] reported that 14 cases (4.7% of the total) required conversion. Research has indicated that the presence of endophytic location, hilar contact, large tumor size, and renal sinus invasion is associated with an increased likelihood of radical surgery [21,22].

A limited number of studies have examined the relationships between intraoperative complications and the MAP and RENAL scores, which are distinct metrics used to evaluate specific tumor characteristics during surgical procedures; in particular, the RENAL score emphasizes tumor complexity, while the MAP score assesses perirenal adhesion. In this context, integrating these two factors can facilitate a comprehensive evaluation of tumor complexity. Several nephrometry scoring systems have been compared to determine their correlation with intraoperative complications [20,21,22]. Nevertheless, only a few studies have considered the MAP score as a potentially significant component. Multivariable analyses identified the MAP score as an independent predictor of the likelihood of requiring radical or open surgical intervention (OR = 7.66; 95% CI 2.67–16.33; *p* < 0.001) [7,11].

The RENAL score showed no significant association with the outcome (OR = 1.31; 95% CI, 0.19–8.78; *p* = 0.78) [23]. Similarly, neither the MAP score nor the RENAL score was predictive of conversion, as demonstrated by Fisher’s exact test (MAP, *p* = 0.16; RENAL, *p* = 0.17) [14,24]. However, both scoring systems exhibited predictive capability for overall intraoperative complications, with all AUC values exceeding 0.5. Notably, the combined use of these scores significantly enhanced predictive performance (*p* < 0.05) [15]. These findings suggest that while the MAP and RENAL scores may have limited utility in predicting conversion or specific operative outcomes, they retain value in anticipating overall intraoperative complications. The improved performance observed with the combined use of both scores highlights the potential benefit of a composite risk assessment approach, rather than reliance on a single anatomical scoring system.

APF may cause damage in local areas, with previous research suggesting that APF might be encountered in 10.6–40.8% of patients [9,10,12,14]. Further studies have shown that higher MAP scores are associated with adverse perioperative outcomes, including longer hospital stays, greater blood loss, and longer operative times [9,12,25]. Higher MAP scores were associated with longer total operating time and dissection duration, as well as increased blood loss [26]. Notably, there were no substantial disparities in warm ischemia time and blood transfusion rates between the groups with low and high MAP scores. The authors attribute the prolonged operative time in the high-MAP group to increased dissection time caused by greater difficulty in peeling off the APF.

Conversely, warm ischemia time is measured from the time of renal artery clamping, when arterial perfusion ceases [26]. The correlation between laparoscopic partial nephrectomy (PN) outcomes and Adherent Perinephric Fat (APF) within a general population was studied in [27], revealing that patients presenting with APF encountered significantly extended surgical durations, increased estimated blood loss, and a greater incidence of renal capsule rupture. Notably, the APF cohort also showed prolonged warm ischemia times. Bylund et al. reported that patients with firmly adherent adipose tissue experienced substantially longer operative times than control patients [13].

In partial nephrectomy, APF was associated with greater estimated blood loss [10]. The MAP score is crucial for predicting the feasibility of segmental artery clamping during laparoscopic PNs [28]. In a single-institution study, Ishiyama et al. found a significant correlation between higher MAP scores and longer dissection times in robot-assisted PNs, including 311 patients [29]. They found a correlation between higher MAP scores and a decreased probability of effective segmental renal artery clamping. Moreover, the MAP score was employed to predict the complexity of robotic PNs [29].

Several studies have shown that combining MAP scores with additional radiographic or nephelometry values improves the prediction of intraoperative and postoperative characteristics. Integrating the MAP and RENAL scores improved the accuracy of predicting total intraoperative issues, surpassing that obtained when either measure was used independently [15]. The RNP score combines elements of the RENAL and MAP scores, and its predictive efficacy for outcomes was assessed [30]. This evaluation focused solely on the tumor radius (R) and proximity to the renal sinus or collecting system (N) components of the RENAL score, as well as the posterior perinephric fat thickness component of the MAP score [30]. It was demonstrated that the RNP score showed strong predictive capacity.

Additionally, there was greater agreement among the observers than with the RENAL score [30]. Tan et al. developed an innovative nomogram to predict intraoperative complications in laparoscopic PNs [31]. Yang et al. considered only the R and N components of the RENAL score [30]; the nomogram also incorporated the perirenal fat stranding component of the MAP score [31]. Based on their research, this new nomogram demonstrated superior predictive accuracy compared with the RENAL and MAP scores when used separately and comparable predictive accuracy when used together, despite containing fewer elements [31].

The limited predictive value observed in this cohort may reflect the inherently lower technical variability and complication risk associated with radical nephrectomy compared with nephron-sparing surgery. As noted, the current study was constrained by a small sample size, which may have restricted the ability to achieve statistical significance. Furthermore, the single-center design restricts the generalizability of the findings. Consequently, we recommend that future longitudinal studies with larger sample sizes and multicenter collaboration be undertaken to validate the results. The inclusion of both open and laparoscopic radical nephrectomies introduces procedural heterogeneity, which may influence operative outcomes and confound associations.

## 5. Limitations

This study has several important limitations that warrant consideration. First, the retrospective single-center design inherently introduces selection bias and limits the generalizability of the findings. Second, the relatively small sample size and the low incidence of Grade IIIb complications significantly diminish statistical power, increase the risk of Type II error, and limit the ability to detect true associations. Furthermore, the low events-per-variable ratio in the regression analysis resulted in broad confidence intervals, indicating model instability and limiting the reliability of multivariable estimation. Accordingly, all regression and ROC analyses should be interpreted as exploratory and hypothesis-generating rather than definitive. Additionally, AUC confidence intervals were estimated using an analytical method rather than resampling approaches, which may limit precision in small samples. Third, the inclusion of both open and laparoscopic radical nephrectomies may have introduced procedural heterogeneity, as the surgical approach itself influences operative time, blood loss, and complication profiles. Furthermore, several potentially significant confounding variables—including body mass index (BMI), the American Society of Anesthesiologists (ASA) score, tumor stage, and surgeon-related factors—were unavailable, which may further limit the interpretation of the results. Fourth, the RENAL and MAP scores were calculated retrospectively from imaging, which may have introduced interobserver variability despite standardized definitions. Although scoring was performed independently by two reviewers with consensus-based resolution, formal interobserver agreement metrics (e.g., kappa or intraclass correlation coefficients) were not assessed, thereby limiting the evaluation of reproducibility. Future studies should incorporate reproducibility analyses to strengthen the methodological robustness of imaging-based scoring systems. Finally, the RENAL and MAP scores were originally developed and validated primarily in nephron-sparing surgery; their applicability to radical nephrectomy, where anatomical complexity and adhesive fat may play a different role, may therefore be limited. Therefore, the findings of this study should be interpreted with caution, as they are exploratory and intended to generate hypotheses.

Further research, including large-scale, multicenter, prospective studies to validate the utility of RENAL and MAP scores in radical nephrectomy procedures, is recommended.

## 6. Conclusions

The RENAL and MAP scores, whether used independently or in combination, did not show a statistically significant predictive association with perioperative outcomes in this small, single-center exploratory cohort. Larger, multicenter prospective studies are necessary to validate these findings.

## Figures and Tables

**Table 1:** Baseline and clinical characteristics of the included patients.

Variable	The Value
Age (Years) (Mean ± SD)	51.64 ± 16.12
Renal Mass Side, N (/%)	
RT	24 (46.2%)
LT	28 (53.8%)
Diabetes Mellitus, N (/%)	
Yes	20 (38.5%)
No	32 (61.5%)
Hypertension, N (/%)	
Yes	26 (50%)
No	26 (50%)
Histopathology, N (%)	
Chromophobe	4 (7.7%)
Clear cell	30 (57.7%)
Clear cell and chromophobe	6 (11.5%)
Invasive urothelial carcinoma	2 (3.8%)
Non-Hodgkin lymphoma	2 (3.8%)
Oncocytoma	2 (3.8%)
Papillary RCC	4 (7.7%)
Squamous cell carcinoma	2 (3.8%)
Tumor Location, N (%)	
Anterior	28 (53.8%)
Posterior	24 (46.2%)
Complications, N (%)	
Yes	6 (11.5)
No	46 (88.5)
Complications According to the Clavien–Dindo Classification System, N (%)	
Grade IIIb	6 (11.5%)
Grade V	2 (3.8%)
No complication	44 (84.6%)
Surgical Procedure, N (%)	
Laparoscopic right radical nephrectomy	14 (26.9%)
Laparoscopic left radical nephrectomy	18 (34.6%)
Left open radical nephrectomy	10 (19.2%)
Right open radical nephrectomy	10 (19.2%)
Operative Time (Hours), Median (IQR)	5 (4–6)
Blood Loss (ml), Median (IQR)	90 (50–500)
Hospital Stays (days), Median (IQR)	5 (4–6)
RENAL Score, Median (IQR)	9 (8–10)
MAP Score, Median (IQR)	3 (2–4)
RENAL Category, N (%)	
Moderate	28 (53.8%)
High	24 (46.2%)
MAP Score Category, N (%)	
Low	12 (23.1%)
Moderate	22 (42.3%)
Moderate	18 (34.6%)

CT: computed tomography; IQR: interquartile range; MAP: Mayo Adhesive Probability.

**Table 2:** Comparison of RENAL and MAP scores according to complications.

Variable		RENAL Score				MAP Score			
		Low N (%)	Moderate N (%)	High N (%)	*p*-Value	Low N (%)	Moderate N (%)	High N (%)	*p*-Value
Complications (Grade IIIb)	Yes	0(0.0)	4(66.7)	2(33.3)	0.674	0(0.0)	2(33.3)	4(66.7)	0.172
	No	0(0.0)	24(52.2)	22(47.8)		12(26.1)	20(43.5)	14(30.4)	

MAP: Mayo Adhesive Probability.

**Table 3:** Spearman’s correlation between RENAL and MAP scores and blood loss, operative time, and length of hospital stay.

Variable	RENAL	*p*-Value	MAP Score	*p*-Value
Blood loss (mL)	−0.114	0.423	0.207	0.141
Operative time (hours)	0.026	0.854	0.211	0.134
Hospital stay (days)	0.294 *	0.035	0.191	0.175

* Correlation is significant at the level of 0.05. MAP: Mayo Adhesive Probability.

**Table 4:** Comparison of operative parameters and scores according to Grade IIIb complications.

Variable	Complications (Grade IIIb)		*p*-Value
	Yes	No	
Operative time (hours) Median (IQR)	5 (4–6)	5 (4–6)	1.000
Hospital stay (days) Median (IQR)	5 (4–6)	5 (3–7)	0.678
RENAL Median (IQR)	8 (8–10)	9 (8–10)	0.304
MAP score Median (IQR)	4 (3–4)	2 (1–4)	0.061

IQR: interquartile range; MAP: Mayo Adhesive Probability.

**Table 5:** Relationship of Grade IIIb complications with age, diabetes mellitus, hypertension, operative time, length of hospital stay, and both scores using binary logistic regression.

	Variable	OR	(95% CI)	*p*-Value
Complications (Grade IIIb)	Age	0.852	(0.714–1.016)	0.075
	Diabetes mellitus	50.38	(0.39–64,773.16)	0.283
	Hypertension	4.068	(0.005–3415.57)	0.683
	Operative time	0.503	(0.105–2.402)	0.389
	Length of hospital stay	0.205	(0.015–2.883)	0.24
	RENAL score	0.858	(0.377–1.954)	0.715
	MAP score	1.721	(0.820–3.615)	0.151

MAP: Mayo Adhesive Probability; OR: odds ratio; CI: confidence interval.

**Table 6:** Comparison of AUC among RENAL score, MAP score, and their combination in the prediction of complications.

Complication (Grade IIIb)	AUC (95% CI)	*p*-Value
RENAL score	0.377 (0.16–0.59)	0.330
MAP score	0.732 (0.52–0.94)	0.067
Combined model	0.638 (0.41–0.86)	0.276

MAP: Mayo Adhesive Probability.

## Data Availability

The data supporting this study’s findings are available from the corresponding author upon reasonable request.
